# Using tocolysis in pregnant women with symptomatic placenta praevia does not significantly improve prenatal, perinatal, neonatal and maternal outcomes: a systematic review and meta-analysis

**DOI:** 10.1186/s13643-018-0923-2

**Published:** 2018-12-27

**Authors:** Frederick Morfaw, Mercy Fundoh, Jessica Bartoszko, Lawrence Mbuagbaw, Lehana Thabane

**Affiliations:** 1 Department of Health Research Methods, Evidence and Impact, McMaster University, Hamilton, Ontario Canada; 2 Department of Obstetrics and Gynaecology, Faculty of Medicines and Biomedical Sciences, University of Yaounde 1, Yaounde, Cameroon; 3 Faculty of Health Sciences, University of Bamenda, Bamenda, Cameroon; 4 Biostatistics Unit, St Joseph’s Healthcare Hamilton, Hamilton, Ontario Canada

**Keywords:** Tocolysis, Tocolytic agent, Placenta praevia, Antepartum haemorrhage

## Abstract

**Background:**

Placenta praevia refers to a placenta located in the lower segment of the uterus. This abnormal location predisposes the placenta to abnormal bleeding with an increased risk of premature labour. The merits of tocolytic drugs (tocolysis) to calm uterine contractions and prolong pregnancy in women with placenta praevia are uncertain.

**Objectives:**

The primary objective is to determine the effects of tocolysis versus no tocolysis on pregnancy prolongation. Secondary objectives include to determining the effects of tocolysis versus no tocolysis on gestational age at delivery, maternal hospitalisations, recurrent vaginal bleeding, prematurity, admissions into neonatology, and perinatal deaths.

**Methods:**

We searched MEDLINE, EMBASE, The Cochrane Central Register of Controlled Trials, reference lists of pertinent articles and trial registries for randomised controlled trials comparing tocolysis to no tocolysis or placebo in patients with placenta praevia. Risk of bias and data extraction was done independently by two reviewers. We pooled data using a random-effects model. We used the GRADE system to assess the certainty of evidence for each outcome.

**Main results:**

There is no significant difference in pregnancy prolongation with the use of tocolysis in cases of placenta praevia (mean difference [MD] 11.51 days; 95% CI, − 1.75, 24.76; 3 trials, 253 participants; low certainty evidence). Tocolysis has no significant effect on gestational age at delivery (MD 0.33 weeks [95% CI − 1.53, 2.19]: 2 trials, 169 participants, moderate certainty evidence), birthweight (MD 0.12 kg [95% CI − 0.26, 0.5 kg]: 2 trials, 169 participants, moderate certainty evidence), risk of premature delivery (risk ratio [RR] 1.04; 95% CI 0.56, 1.94): 2 trials, 169 participants, low certainty evidence), risk of repeat vaginal bleeding (RR 1.05 [95% CI 0.73, 1.51]: 2 trials, 169 participants, moderate certainty evidence). Tocolysis has no significant effect on the risk of perinatal death (risk difference [RD]: 0.00 [95% CI − 0.04, 0.03]: 2 trials, 169 women; low certainty evidence), number of days of maternal hospitalisation (MD 0.60 days [95% CI − 0.79, 1.99]: 1 trial, 109 women; low certainty evidence), risk of fetal admissions into neonatology (RR 1.30 [95% CI 0.80, 2.12]: 1 trial, 109 participants, low certainty evidence) and on the duration of stay in neonatology units (MD 0.70 days [95% CI − 5.26, 6.66]: 1 trial, 109 participants, low certainty evidence).

**Conclusion:**

In women with symptomatic placenta praevia, there is no significant effect on pregnancy prolongation with the use of tocolysis. Tocolysis has no significant effect on other prenatal, perinatal, neonatal and maternal outcomes among women with symptomatic placenta praevia.

**Systematic review registration:**

PROSPERO CRD42018091513

**Electronic supplementary material:**

The online version of this article (10.1186/s13643-018-0923-2) contains supplementary material, which is available to authorized users.

## Background

### Description of the condition

Placenta praevia is a clinical condition in pregnancy in which the placenta is implanted on or near the internal os of the uterine cervix [1]. Given this unusually low implantation of the placenta in the uterus, there is a risk of severe or repeated vaginal bleeding before delivery. The prevalence of placenta praevia is about 3 to 5 cases per 1000 singleton pregnancies [1, 2]. Placenta praevia may have serious maternal and fetal consequences [3]. Fetal consequences of placenta praevia include severe prematurity leading to increased perinatal mortality [4]. There is an estimated three to fourfold increase in perinatal mortality in cases of prematurity following placenta praevia, with rates of perinatal mortality in this group being estimated at about 10 to 12/1000 births [5, 6]. The maternal consequences of placenta praevia include maternal death, with a maternal mortality rate of 0.03% in developed countries [7].

### The intervention

Tocolytic or anticontraction drugs are a class of drugs which act by suppressing uterine contractions and thereby preventing premature labour. They include beta adrenergic agonists, calcium channel blockers, magnesium sulfate, oxytocin receptor antagonist, progesterone, prostaglandin synthesis inhibitors, and nitrogen oxide [8]. Among certain patients with placenta praevia, there are clinically observable uterine contractions [4]. Furthermore, other studies have reported the existence of subclinical uterine contractions prior to bleeding in cases of haemorrhagic placenta praevia [9, 10]. These uterine contractions are usually associated with vaginal bleeding [11, 12]. It is therefore plausible to justify the use of drugs which suppress uterine contractions in cases of symptomatic placenta praevia.

### How does the intervention work?

These drugs are used in selected cases of placenta praevia to reduce uterine contractions secondary to bleeding and also to decrease the bleeding episodes linked to uterine activity [13]. Beta adrenergic receptor agonists such as ritodrine and salbutamol work by inhibiting intracellular cyclic AMP concentration, thereby enhancing relaxation of the myometrium [14]. Calcium channel blockers on the other hand decrease intracellular free calcium concentration, thereby inducing myometrial relaxation [15]. Progesterone acts by directly regulating calcium concentration within myometrial cells and by regulating the synthesis of prostaglandins [15]. Magnesium sulfate decreases intracellular calcium concentration, thereby blocking contraction [15]. Oxytocin receptor agonists block intracytoplasmic calcium release which is responsible for contractions and reduces the synthesis of prostaglandins [15].

### Why is it important to do this review?

The use of tocolytic agents in symptomatic placenta praevia is controversial [16]. Tocolytic use in the management of symptomatic placenta praevia has been described as anecdotal [11, 17], and while it is recommended as part of the conservative management of placenta praevia in certain settings [18], in other settings, it is not routinely used [13]. In addition, there are safety concerns regarding the potential side effects of the drugs [13].

Evidence of the clinical utility of tocolytic use in prolonging pregnancy and reducing prematurity is limited. A preliminary literature search on this subject identified just one review by Bose et al. in 2011 [1]. They included 02 retrospective studies [16, 19] and 01 randomised controlled trial (RCT) [13]. The lone RCT by Sharma et al. [13] concluded on the prolongation of pregnancy with the use of tocolysis. However, the combined result did not confirm this. Given that this conclusion was not based on summary evidence of RCTs, we believe a summary evidence of RCTs will provide a higher quality evidence for the use or non-use of tocolysis in cases of symptomatic placenta praevia.

## Objectives

Our primary objective was to determine the effects of tocolysis compared to no tocolysis on pregnancy prolongation among pregnant women with symptomatic placenta praevia. Our secondary objectives were to determine the effects of tocolysis compared to no tocolysis on gestational age at delivery, maternal hospitalisations, recurrent vaginal bleeding, prematurity, admissions into neonatology and perinatal deaths among pregnant women with symptomatic placenta praevia.

## Methods

This systematic review was registered with the international prospective register of systematic reviews (PROSPERO), registration number CRD42018091513.

### Criteria for considering studies for this review

#### Types of studies

We included randomised clinical trials (RCTs) that evaluated the use of any tocolytic treatment compared to no tocolysis in prolonging the duration of pregnancy among women with symptomatic placenta praevia.

#### Types of participants

Our study population included all pregnant women with pregnancies ranging between 15 weeks and 36 weeks gestational age who presented to the hospital with per vaginal bleeding with intact membranes, and in whom a clinical diagnosis of placenta praevia was confirmed by the attending physician following a transabdominal ultrasound. The gestational ages were calculated from the first day of the last menstrual period, or from a first trimester routine ultrasound by measuring the crown rump length. Any studies with only a subset of the relevant participants were included, and data was specifically abstracted only for this subset of women. The gestational age range between 15 to 36 weeks was chosen as it represented the range of gestational ages used in defining cases of placenta praevia [20].

We excluded studies on women with severe antepartum haemorrhage necessitating immediate delivery, those with preeclampsia or eclampsia, women with placental abruption, acute fetal distress, intra uterine death, chorioamnionitis, cardiopathy in pregnancy, and those with any known contraindications to tocolytic drugs. We also excluded studies with patients who received strategies such as cervical stitch which could potentially alter the duration of pregnancy.

#### Types of interventions

We assessed any tocolytic therapy applied orally or parenterally to a woman specifically because of a perceived likelihood that this therapy would prolong the duration of pregnancy, reduce uterine activity or reduce vaginal bleeding. Tocolytic therapies considered included all beta adrenergic drugs, all calcium channel blockers, magnesium sulfate, progesterone, oxytocin inhibitors, prostaglandin synthesis inhibitors, nitroglycerin and non-steroidal anti-inflammatory drugs which are the known tocolytics in clinical use [8]. The above drugs were considered irrespective of the dose, frequency, timing, duration or hospital admission. In cases where the tocolytic drugs were combined, these patients were considered as having received tocolysis in general and were analysed as such. In each case, the intervention was associated with standard care comprising of bed rest and the administration of corticosteroids to accelerate fetal lung maturity where indicated.

#### Comparisons

The control groups were expected to receive no form of tocolysis, but rather standard of care including strict bed rest and reduced physical activity, with or without steroid prophylaxis for accelerated fetal lung maturity. Placebo treatments were also eligible.

#### Outcomes

The maternal outcomes were: duration of pregnancy prolongation in days from the time of admission until delivery, the number of days of maternal hospitalisation and any repeat episodes of vaginal bleeding. The fetal outcomes included mean birth weight at delivery in kilogrammes, mean gestational age at delivery in weeks, premature delivery (before 37-week gestation), admissions in neonatology units, duration of stay in neonatology units in days and perinatal deaths. These outcomes reflect the desired goals when tocolytics are employed in this indication.

For the main outcome of days of pregnancy prolongation with tocolysis, our minimum important difference was 7 days. This was based on a survey of Canadian obstetricians 70% of whom reported at least a 1 week minimal clinically important treatment effect needed to change practice in preterm birth prevention [21].

#### Electronic searches

Using the OVID search platform, we searched MEDLINE and EMBASE from inception to January 2018, and the Cochrane Central Register of Controlled Trials (CENTRAL) through the Cochrane Library (Issue 12 of 12, December 2017). We set no limitations on language or the publication status of the studies. (See Additional file 1: Appendix 1 for the MEDLINE search strategy).

#### Searching other resources

We hand-searched the reference list of relevant reviews and clinical trials identified through the electronic searches. We searched for grey literature as well as the World Health Organization International Clinical Trials Registry Platform (http://apps.who.int/trialsearch/), in order to identify ongoing trials or completed but unpublished trials. Finally, we contacted experts in the field by email for any ongoing studies or relevant but unpublished studies.

### Data collection and analysis

#### Study selection

**Fig. 1:**
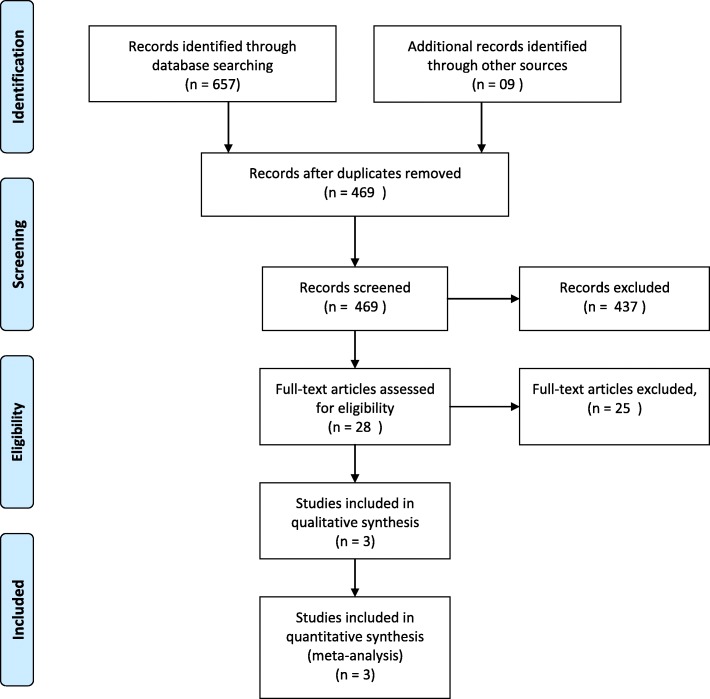
PRISMA flow diagram

Two review authors (FM and MF) independently screened the titles and abstracts of the studies identified through the electronic searches in order to identify possible articles for inclusion. Following this screening, the full texts of eligible articles were obtained and assessed based on our inclusion criteria cited above. Discrepancies were resolved by discussion or consultation with a third author (JB). We constructed a PRISMA flow diagram (Fig. 1) describing the study selection process. We provided a list of the excluded studies with a reason for their exclusion in the ‘Characteristics of excluded studies’ (Additional file 1: Appendix 2).

#### Data extraction and management

Data was extracted using a pre-designed and pre-tested Microsoft Excel data extraction form. The data extraction was done by the two authors (FM and MF) working independently of each other. Data was extracted on the design, the sample size, the participant characteristics, the interventions and the different outcomes of interest in each group. In cases of data reported solely as graphs, review authors were contacted for the raw data, and where not possible, extrapolations were made directly from the graphs. Discrepancies were resolved either by discussion or by consultation with a third author (JB). In cases of incomplete data, missing data or uncertainty, we contacted the authors of the principal trials by email for clarifications.

#### Assessment of risk of bias in included studies

We used the criteria outlined in the Cochrane Handbook for Systematic Reviews of Interventions [22] to assess the risk of bias in studies included in the review. Using these criteria, we assessed the following domains: random sequence generation, allocation concealment, blinding of participants and personnel, blinding of outcome assessment, incomplete outcome data, selective reporting and other sources of bias. The risk of bias table was completed for each outcome by two review authors working independently of each other. Studies were rated as being at either ‘high’, ‘low’ or ‘unclear’ risk of bias. As much as possible, we avoided the term ‘unclear’ in describing the risk of bias, except in the rare situations when the review authors could not make any judgement.

#### Measures of treatment effect

Statistical analyses were done using Review Manager 5 [23]. The unit of analysis was the individual. We used mean differences, relative risks and risk difference to measure the effect of the intervention between the two groups.

#### Assessment of heterogeneity

We assessed clinical heterogeneity in the included studies by assessing the patients (pregnant women with confirmed placenta praevia), the intervention (any form of tocolytic therapy) and the primary outcome (duration of pregnancy prolongation), to see if they were sufficiently similar to be pooled together. We then evaluated statistical heterogeneity***.*** This was done by a combination of visual inspection of the confidence interval of pooled trials for any overlap, the chi^2^ test of homogeneity (statistical significance threshold *P* < 0.10) and the *I*^2^ statistic. We rated statistical heterogeneity based on the *I*^2^ statistic as described in the Cochrane Handbook of Systematic reviews [22].

#### Data synthesis

Review Manager Software (RevMan 5) was used to meta-analyse results from the included trials. Meta-analysis was conducted using a random-effects model. We computed the risk ratio (RR) for binary data, and the mean difference (MD) for continuous data measured on the same scale. In cases where one or more events occurred in one group and none in the other group, we used the risk difference (RD) as a measure of treatment effect as a risk ratio could not be computed in such cases.

#### Subgroup analysis

We planned a subgroup analysis comparing those with single tocolysis used versus those with one or more tocolytic drugs combined, against the control group. This was however not conducted because of lack of sufficient number of studies with the above subgroups.

#### Sensitivity analysis

We conducted a sensitivity analysis excluding studies judged to be at high risk of bias overall, in order to determine the robustness of our conclusions.

#### Quality of the evidence

We evaluated the overall quality of the evidence for each of the key outcomes using the Grading of Recommendations Assessment, Development and Evaluation (GRADE) system for grading evidence described by Guyatt et al. [24]. We used the online GRADEpro Guideline Development Tool (GDT) [25] to construct a ‘Summary of findings’ table for our review (Additional file 1: Appendix 4).

## Results

### Description of the search

We screened a total of 469 records after removal of duplicates from MEDLINE (90), EMBASE (369) and the Cochrane Central Register of Controlled Trials (CENTRAL) (09). No additional records were identified from other sources after removal of duplicates.

We identified 28 studies that could potentially be included in the review and whose full texts were assessed for eligibility. Following full-text eligibility screening, three studies were retained for inclusion in the review and were meta-analysed. The PRISMA diagram (Fig. 1) summarises the process of screening and selecting studies for inclusion in the review.

### Included studies

Additional file 1: Appendix 3 (characteristics of included studies) summarises the key features of the included studies. One of the studies was a multicentre study conducted in five centres in France [4], while the other two were single site studies conducted in India [13] and Poland [26]. The three studies included a total of 253 pregnant women. Two classes of tocolytic drugs were used in the three studies, with Sharma et al. [13] and Sozanski et al. [26], using β2-adrenoreceptor agonists (ritodrine and fenoterol, respectively) while Verspyck et al. [4] used the calcium channel blocker nifedipine. The use of a matching placebo was employed only by one study [4]. In all three studies, both study groups received the same standard of care and differed only in the intervention of interest.

### Excluded studies

Additional file 1: Appendix 2 (characteristics of excluded studies) contains a list of the excluded studies together with the reasons for their exclusion.

### Risk of bias in included studies

**Fig. 2:**
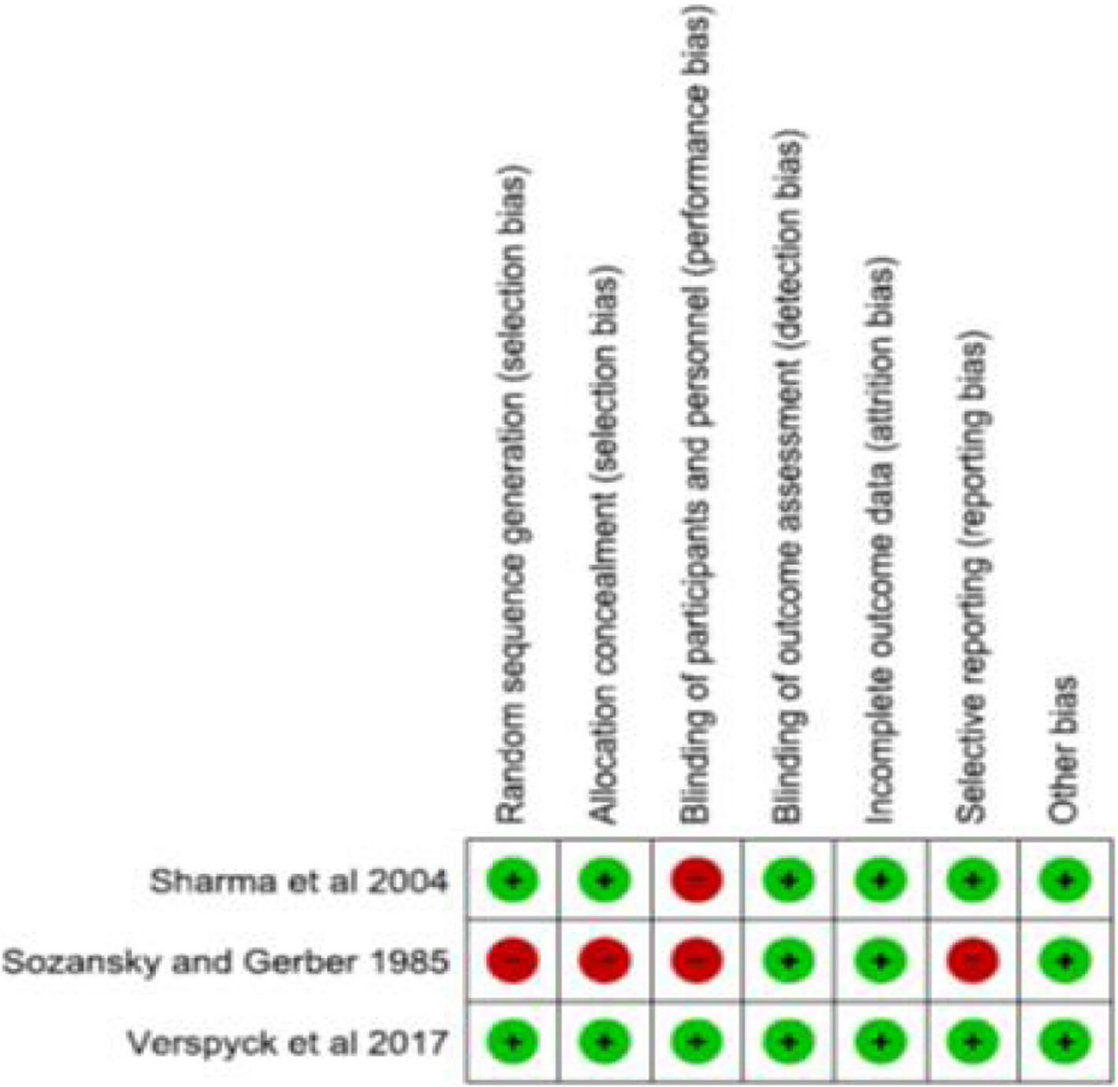
Risk of bias summary

We included an assessment of the risk of bias of each of the individual studies in the characteristics of included studies table (Additional file 1: Appendix 3). Figure 2 summarises the risk of bias of each study individually. In order to exclude the risk of bias due to selective outcome reporting, we checked for trial registrations and protocols in order to verify if the intended outcomes were actually the outcomes that were reported. The trial by Verspyck et al. [4] was registered with ClinicalTrials.gov (registration number NCT00620724), and all outcomes were pre-specified and reported as planned in the study (low risk of reporting bias). The trial by Sharma et al. [13] was not registered and a protocol for the study was not available. However, all expected outcomes identified in the methods are reported as planned by the study. Given the overall aim of tocolysis in pregnancy is to arrest uterine contractions, thereby prolonging the duration of pregnancy [27], the risk of selective outcome reporting for this primary efficacy outcome is low. The trial by Sozanski et al. [26] was not registered, and no protocol available to compare pre-specified outcomes with those reported. This trial only reported on 1 outcome: pregnancy prolongation, thus increasing the risk of selective outcome reporting in this study.

### Effects of intervention

A ‘summary of findings’ tables for the main comparisons done can be found in Additional file 1: Appendix 4.

### Duration of pregnancy prolongation

**Fig. 3:**
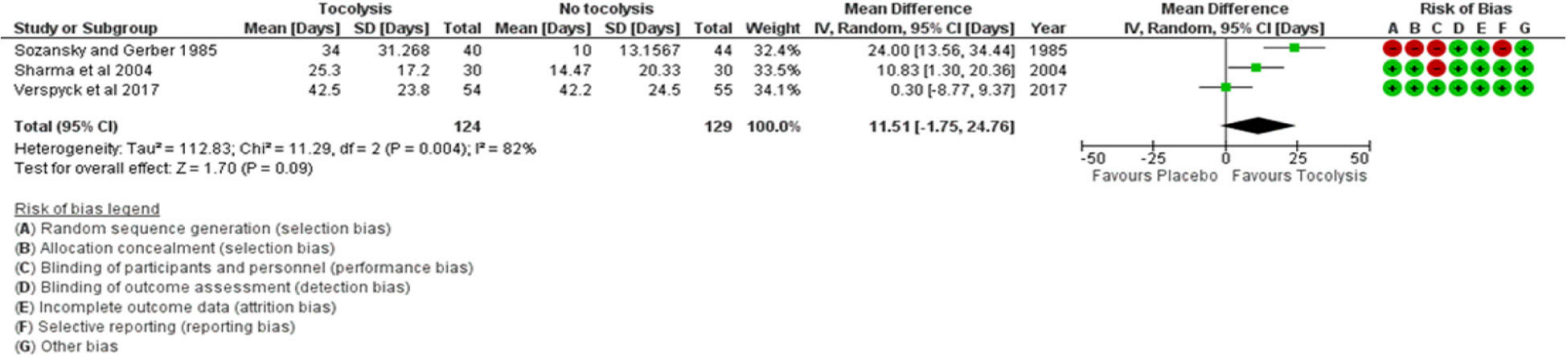
Comparison: provision of tocolysis versus placebo. Outcome: mean difference in number of days of pregnancy prolongation

There was no significant difference in mean days of pregnancy prolongation between the women who received tocolysis and those who did not (mean difference [MD] 11.51 more days; 95% CI − 1.75 to 24.76; 3 trials, 253 participants; *I*^2^ = 82%; low certainty evidence) (see Fig. 3).

### Mean gestational age at delivery

**Fig. 4:**
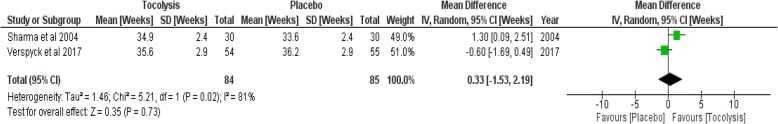
Comparison: provision of tocolysis versus placebo. Outcome: mean difference in gestational age at delivery

There was no significant difference in mean gestational age at delivery between the women who received tocolysis and those who did not (mean difference [MD] 0.33 more weeks; 95% CI − 1.53, 2.19; 2 trials, 169 participants; *I*^2^ = 82%; moderate certainty evidence) (see Fig. 4).

### Mean birth weight

**Fig. 5:**
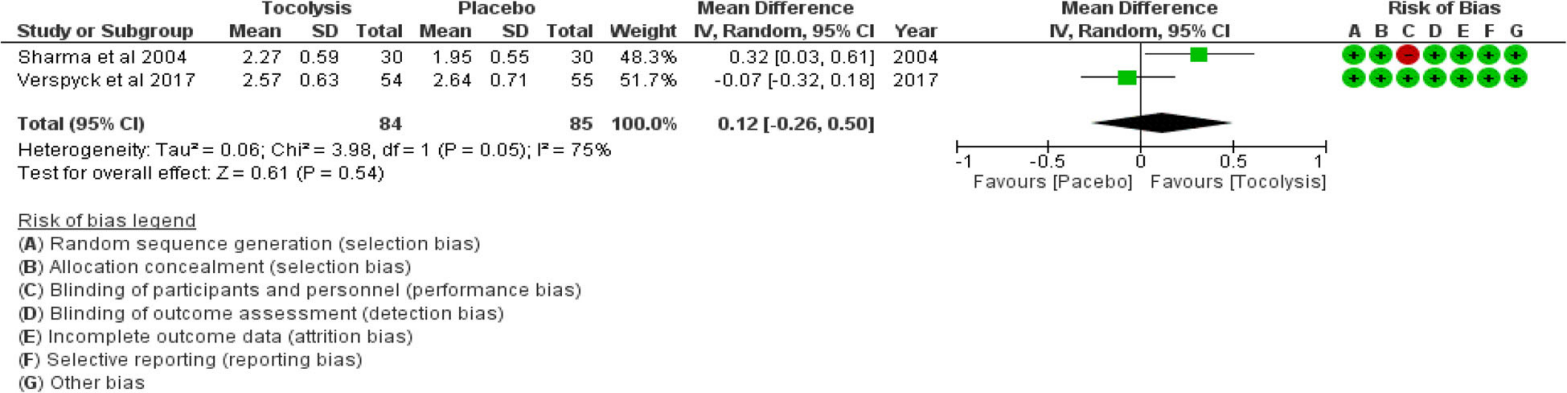
Comparison: provision of tocolysis versus placebo. Outcome: mean difference in birth weight

There was no significant difference in mean birth weight between the women who received tocolysis and those who did not (mean difference [MD] 0.12 kg more; 95% CI − 0.26, 0.5; 2 trials, 169 participants; *I*^2^ = 75%; moderate certainty evidence)***.*** (see Fig. 5).

### Premature deliveries

**Fig. 6:**
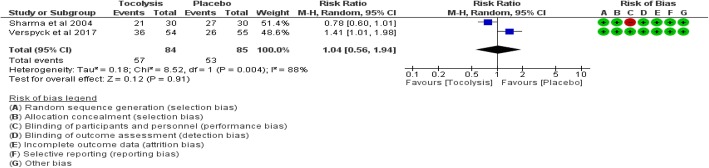
Comparison: provision of tocolysis versus placebo. Outcome: relative risk of premature delivery

Two studies (169 women) contributed data for this outcome. Using tocolysis versus not using it had no significant effect on the risk of a premature (RR 1.04; 95% CI 0.56, 1.94). Statistical heterogeneity was substantial (*I*^2^ = 88%) (moderate certainty evidence) (see Fig. 6).

### Repeat episodes of vaginal bleeding

**Fig. 7:**
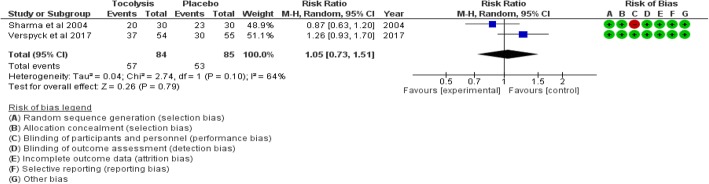
Comparison: provision of tocolysis versus placebo. Outcome: relative risk of any repeat episode of vaginal bleeding

Two studies (169 women) contributed data for this outcome. Using tocolysis versus not using it had no significant effect on the risk of any repeat episode of vaginal bleeding (RR 1.05 [95% CI 0.73, 1.51]). Statistical heterogeneity was substantial (*I*^2^ = 64%) (low certainty evidence) (see Fig. 7).

### Perinatal deaths

**Fig. 8:**
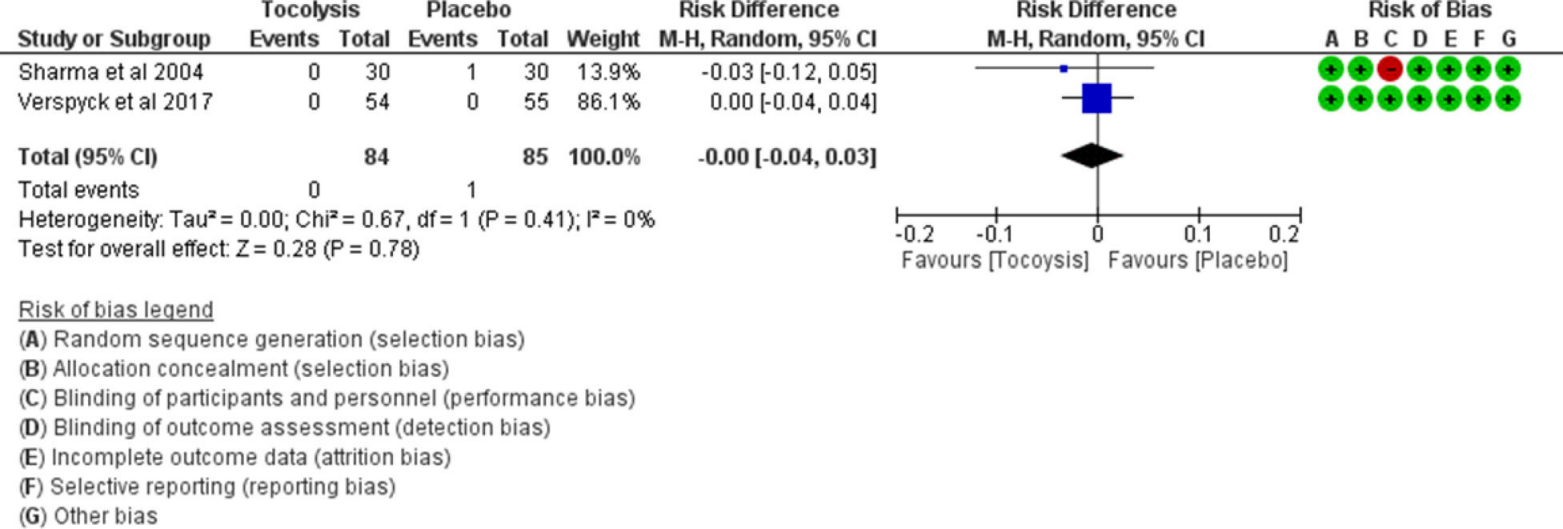
Comparison: provision of tocolysis versus placebo. Outcome: risk difference in perinatal deaths

Two studies (169 women) contributed data for this outcome. Only one perinatal death was recorded in one of the trials. We therefore used the risk difference as a summary measure to estimate the effect of the intervention. There was no significant risk difference of perinatal death between tocolysis versus no tocolysis (RD 0.00 [95% CI − 0.04, 0.03]). Statistical heterogeneity across the studies included in this outcome might not be important (*I*^2^ = 0%) (low certainty evidence) (see Fig. 8).

### Number of days of maternal hospitalisation

Only one study [4] reported on this outcome (109 women), so a meta-analysis was not conducted. The mean difference in duration of maternal hospitalisation was 0.60 days [95% CI − 0.79, 1.99] with tocolysis versus no tocolysis (low-quality evidence).

### Fetal admissions into neonatology

Only one study [4] reported on this outcome (109 women), so a meta-analysis was not conducted. Using tocolysis versus not using it had no significant effect on the risk of the newborn being admitted in neonatology units (RR 1.30 [95% CI 0.80, 2.12]) (low certainty evidence).

### Duration of stay in neonatology units

Only one study [4] reported on this outcome (109 women), so a meta-analysis was not conducted. The mean difference in duration of stay in neonatology units following the intervention was 0.70 more days [95% CI − 5.26, 6.66] with tocolysis versus no tocolysis (low certainty evidence).

### Sensitivity analyses

The result for the outcome on pregnancy prolongation was similar when we excluded the studies at high risk of bias (See Additional file 1: Appendix 5).

## Discussion

### Summary of main results

There is no significant difference in pregnancy prolongation with the use of tocolysis in cases of placenta praevia (mean difference [MD] 11.51 days; 95% CI, − 1.75, 24.76; 3 trials, 253 participants; low certainty evidence). Tocolysis has no significant effect on gestational age at delivery (MD 0.33 weeks [95% CI − 1.53, 2.19]: 2 trials, 169 participants, moderate certainty evidence), birthweight (MD 0.12 kg [95% CI − 0.26, 0.5 kg]: 2 trials, 169 participants, moderate certainty evidence), risk of premature delivery (risk ratio [RR] 1.04; 95% CI 0.56, 1.94): 2 trials, 169 participants, low certainty evidence), risk of repeat vaginal bleeding (RR 1.05 [95% CI 0.73, 1.51]: 2 trials, 169 participants, moderate certainty evidence). Tocolysis has no significant effect on the risk of perinatal death (risk difference [RD]: 0.00 [95% CI − 0.04, 0.03]: 2 trials, 169 women; low certainty evidence), number of days of maternal hospitalisation (MD 0.60 days [95% CI − 0.79, 1.99]: 1 trial, 109 women; low certainty evidence), risk of fetal admissions into neonatology (RR 1.30 [95% CI 0.80, 2.12]: 1 trial, 109 participants, low certainty evidence) and on the duration of stay in neonatology units (MD 0.70 days [95% CI − 5.26, 6.66]: 1 trial, 109 participants, low certainty evidence). A sensitivity analysis without the trial by Sozanski et al. [26] (considered at high risk of bias for the outcome of duration of pregnancy prolongation) did not make any difference on the overall conclusion for this outcome.

### Agreement and disagreement with other reviews

We identified just one previous review reporting on the use of tocolysis in patients with symptomatic placenta praevia [1]. This review included two observational studies [16, 19] and one RCT [4] (which was also included in this review). One of the studies included in our review was published in Polish [26] and not included in the previous review.

Bose et al. [1] had three main findings against which our results are compared. They concluded on the limited number of publications on the management of symptomatic placenta praevia, a fact which is upheld by our study 7 years later. They did a narrative synthesis of the evidence and concluded on the fact that the use of tocolysis prolongs pregnancy by more than 7 days in cases of symptomatic placenta praevia [1]. Other reports from observational studies are in favour of pregnancy prolongation with the use of tocolysis in cases of placenta praevia [12, 28–30]. These findings contrast with our findings which suggest that there is no significant difference in pregnancy prolongation with the use of tocolysis in cases of placenta praevia. Thirdly, the review by Bose et al. [1] highlighted the limited information on materno-fetal outcomes with the use of tocolysis in cases of placenta praevia. This is equally highlighted in our study and points towards the overall research gap on the use of tocolysis in cases of placenta praevia.

The fundamental difference between this review and that of Bose et al. [1] is their use of observational studies and its inherent biases due to the lack of randomisation which may lead to differential effect estimates. Their overall conclusion on the limited/cautious use of tocolytics in women with symptomatic placenta praevia as recommended by the Royal College of Obstetricians and Gynaecologists [31] only partially aligns with our results which shows there is no difference in maternal and fetal outcomes when tocolysis is used in cases of symptomatic placenta praevia.

### Strengths of this review

In order to minimise bias in our review, we employed a comprehensive literature search of major databases and grey literature over a large period of time. We also contacted experts in the field for ongoing or unpublished reports, and we did not limit our search by language or any search filters. Two independent authors working remotely from each other conducted the study identification inclusion, data extraction and risk of bias assessments.

### Potential biases in the review

Publication bias was undetected but cannot be excluded. In one study [26], results were extrapolated from a graph as the actual figures were not reported by the authors.

### Overall completeness and applicability of the evidence

Two of the trials included were conducted in Europe and one in Asia. We are confident that the right participants, interventions (all possible tocolytics), comparisons and outcomes have been explored with this evidence. Given that these trials were conducted in settings where surveillance of patients under tocolysis is supposedly optimal, it may limit the applicability of the findings especially in underdeveloped settings where this is not often the case.

### Quality of the evidence

Overall, our judgement of the quality of evidence for the outcomes in this review is moderate to low. This is primarily due to risk of bias among the included studies and imprecision of the results mostly linked to the total number of events that occurred across studies and the wide confidence intervals of our estimates.

## Conclusions

### Implications for clinical practice

Based on our results and the quality of the evidence, clinicians wishing to administer tocolysis in cases of placenta praevia should bear in mind that it may result in little to no difference on pregnancy prolongation and other maternal and perinatal outcomes.

### Implications for policy

There is a need to reconsider current policies on the use of tocolysis in cases of symptomatic placenta praevia in the light of present day evidence. In particular, policies recommending the cautious use of tocolysis in cases of placenta praevia need to be reconsidered as there may not be enough evidenced to recommend its use in this indication.

### Implications for research

The present body of evidence on the use of tocolysis in cases of symptomatic placenta praevia is of low quality, hence larger clinical trials are necessary to improve upon the quality of the evidence. Our review highlights a research gap on tocolysis in cases of placenta praevia on outcomes such as maternal mortality, post-partum haemorrhage and the risk of blood transfusions which are of direct relevance in underdeveloped settings and not evaluated in this review. A future research question is therefore to determine the effects of tocolysis compared to no tocolysis on maternal mortality, post-partum haemorrhage and the risk of blood transfusion among pregnant women with symptomatic placenta praevia.

## Additional file

Additional file 1: Appendices 1–5 (DOCX 222 kb)

## Abbreviations

CENTRAL: Cochrane Central Register of Controlled Trials
CI: Confidence interval
cyclic AMP: Cyclic adenosine monophosphate
EMBASE: Excerpta Medica dataBASE
GDT: Guideline Development Tool
GRADE: Grading of Recommendations Assessment, Development and Evaluation
MD: Mean difference
MEDLINE: Medical Literature Analysis and Retrieval System Online
PRISMA: Preferred Reporting Items for Systematic Reviews and Meta-Analyses
PROSPERO: International prospective register of systematic reviews
RCT: Randomised controlled trial
RD: Risk difference
RR: Risk ratio
