# Supplementary material for: Using tocolysis in pregnant women with symptomatic placenta praevia does not significantly improve prenatal, perinatal, neonatal and maternal outcomes: a systematic review and meta-analysis
Source: Syst Rev. 2018 Dec 27;7:249. doi: 10.1186/s13643-018-0923-2 (PMC6307312; doi:10.1186/s13643-018-0923-2)
Supplement: Supplementary file 1 — Appendices 1–5 (DOCX 222 kb) [file 13643_2018_923_MOESM1_ESM.docx]

**Appendix 1 MEDLINE search strategy 26 January 2018**

Database(s): OVID Medline Epub Ahead of Print, In-Process & Other Non-Indexed Citations, Ovid MEDLINE(R) Daily and Ovid MEDLINE(R) 1946 to Present 
Search Strategy:

| **#** | **Searches** | **Results** |
| --- | --- | --- |
| 1 | placenta previa.mp. or exp Placenta Previa/ | 3610 |
| 2 | placenta praevia.mp. | 1388 |
| 3 | placenta pr?evia.mp. | 4017 |
| 4 | antepartum h?emorrhage.mp. | 729 |
| 5 | low lying placenta.mp. [mp=title, abstract, original title, name of substance word, subject heading word, keyword heading word, protocol supplementary concept word, rare disease supplementary concept word, unique identifier, synonyms] | 124 |
| 6 | lower uterine segment placenta.mp. | 3 |
| 7 | (placenta adj3 (h?emorrhag* or bleed* or pr?evia or lower uterine segment)).mp. [mp=title, abstract, original title, name of substance word, subject heading word, keyword heading word, protocol supplementary concept word, rare disease supplementary concept word, unique identifier, synonyms] | 4281 |
| 8 | or/1-7 | 4909 |
| 9 | tocolysis.mp. or exp TOCOLYSIS/ or exp Adrenergic beta-Agonists/ or exp Fenoterol/ | 113317 |
| 10 | tocoly$.mp. | 4141 |
| 11 | tocolytic agent.mp. or exp Tocolytic Agents/ | 63913 |
| 12 | calcium-channel blocker$.mp. | 43141 |
| 13 | oxytocin inhibitor$.mp. | 16 |
| 14 | oxytocin antagonist$.mp. | 448 |
| 15 | betamimetic$.mp. | 251 |
| 16 | betasympathomimetic$.mp. | 17 |
| 17 | betaadrenergic$.mp. | 53 |
| 18 | cyclo-oxygenase-2 inhibitor$.mp. | 312 |
| 19 | cox 2 inhibitors$.mp. | 3903 |
| 20 | exp PROGESTERONE/ | 67546 |
| 21 | progesterone.mp. | 102468 |
| 22 | progestative.mp. | 198 |
| 23 | Albuterol.mp. | 10155 |
| 24 | Salbutamol.mp. | 6997 |
| 25 | Fenoterol.mp. | 2105 |
| 26 | Hexoprenaline.mp. | 190 |
| 27 | Indomethacin.mp. | 41392 |
| 28 | Isoxsuprin.mp. | 10 |
| 29 | magnesium sulfate.mp. | 6231 |
| 30 | magnesium sulphate.mp. | 1461 |
| 31 | Nifedipine.mp. | 22814 |
| 32 | Nylidrin.mp. | 186 |
| 33 | orciprenaline.mp. | 425 |
| 34 | Ritodrine.mp. | 1151 |
| 35 | Terbutaline.mp. | 4003 |
| 36 | Nicardipine.mp. | 3875 |
| 37 | Atosiban.mp. | 420 |
| 38 | celecoxib.mp. | 6054 |
| 39 | nitroglycerin.mp. | 15350 |
| 40 | or/9-39 | 352662 |
| 41 | 8 and 40 | 90 |

**Appendix 2 excluded studies with reasons**

| **Article** | **Reason for exclusion** |
| --- | --- |
| Crowther et al 2017 (1) | Wrong study population. No placenta praevia |
| Norman et al 2016 (2) | Wrong study population. No placenta praevia |
| The pregnancy meeting(3) | Wrong article type, conference proceeding report |
| Shirazi and Ul Huda Ansari 2015 (4) | Wrong study population No placenta praevia |
| Opmeer et al 2011 (5) | Wrong study population: No placenta praevia |
| Marret et al 2007 (6) | Wrong outcomes : evaluated tocolysis on neuroprotection in preterm infants |
| Maintenance tocolysis between clinical practice and evidence-based medicine 2007 (7) | Wrong design: Opinion paper |
| Jaswal 2006 (8) | Wrong intervention (cerclage) |
| Carbillon 2004 (9) | Wrong study design This was a scoping review article of tocolytic use in multiple pregnancy |
| Towers et al 1999 (10) | Wrong study design: This was a retrospective analysis |
| Besinger et al 1995 (11) | Wrong study design: This was a retrospective analysis |
| Watson et al 1990 (12) | Wrong study design: This was a retrospective analysis |
| Saller et al 1990 (13) | Wrong study design: This was a retrospective analysis |
| Celayeta 1988 (14) | This was a comment on the results on another published study on the use of tocolysis in placenta praevia |
| Tomich 1985 (15) | Wrong design, case series with no comparison group |
| Nakamura et al 1984 (16) | Wrong design, case series with no comparison group |
| Sampson et al 1984 (17) | Wrong design, case series with no comparison group |
| Flowers et al (18) | Wrong design. This was a case report of the use of ritodrine in the management of placenta praevia in a 25 year old patient. No comparison group. |
| Hastwell et al 1979 (19) | Wrong design. This was a retrospective analysis comparing tocolysis vs no tocolysis in cases of placenta praevia |
| Duran Sanchez 1975 (20) | Wrong design: Review article |
| Mosler et al 1974 (21) | Wrong design: Review article |
| Casati et al 1973 (22) | Wrong design, case series with no comparison group |
| Bose et al 2011 (23) | Wrong design: Review article |
| Lu and Guo 1998 (24) | Wrong comparator (magnesium sulfate which is another tocolytic) |
| Besinger et al 1995 (25) | Wrong design: This was a retrospective analysis |
|  |  |

References

1. C.A. C, P. A, A.J. M, V. F, T. T, J.M. D, et al. Vaginal progesterone pessaries for pregnant women with a previous preterm birth to prevent neonatal respiratory distress syndrome (the PROGRESS Study): A multicentre, randomised, placebo-controlled trial. PLoS Med [Internet]. 2017;14(9):e1002390. Available from: http://medicine.plosjournals.org/perlserv/?request=index-html&issn=1549-1676

2. J.E. N, N. M, C.-M. M, A. S, P.R. B, S. T, et al. Vaginal progesterone prophylaxis for preterm birth (the OPPTIMUM study): A multicentre, randomised, double-blind trial. Lancet [Internet]. 2016;387(10033):2106–16. Available from: http://www.journals.elsevier.com/the-lancet/

3. Anonymous. 35th Annual Meeting of the Society for Maternal-Fetal Medicine: The Pregnancy Meeting. Am J Obstet Gynecol [Internet]. 2015;212(1 SUPPL. 1). Available from: http://ovidsp.ovid.com/ovidweb.cgi?T=JS&PAGE=reference&D=emed17&NEWS=N&AN=71743335

4. A. S, N. UHA. Comparison of efficacy for tocolysis of preterm labour between magnesium sulphate and nifedipine. Pakistan J Med Heal Sci [Internet]. 2015;9(4):1177–80. Available from: http://pjmhsonline.com/2015/oct_dec/pdf/1177 Comparison of Efficacy for Tocolysis of Preterm Labour between Magnesium Sulphate and Nifedipine.pdf

5. B. O, S. V, B.W. M, J. VDP, J. K, D. P, et al. Assessment of perinatal outcome with sustained tocolysis in early labor (APOSTEL). Am J Obstet Gynecol [Internet]. 2011;204(1 SUPPL.):S2. Available from: http://ovidsp.ovid.com/ovidweb.cgi?T=JS&PAGE=reference&D=emed13&NEWS=N&AN=70328015

6. S. M, L. M, V. Z-S, D. E, C. L, M.-F. H. Magnesium sulphate given before very-preterm birth to protect infant brain: The randomised controlled PREMAG trial. BJOG An Int J Obstet Gynaecol [Internet]. 2007;114(3):310–8. Available from: http://ovidsp.ovid.com/ovidweb.cgi?T=JS&PAGE=reference&D=emed11&NEWS=N&AN=46256042

7. Maintenance tocolysis between clinical practice and evidence-based medicine. Geburtshilfe Frauenheilkd [Internet]. 2007;67(1):28–32. Available from: http://ovidsp.ovid.com/ovidweb.cgi?T=JS&PAGE=reference&D=emed11&NEWS=N&AN=46382043

8. Jaswal A, Manaktala U, Sharma JB. Cervical cerclage in expectant management of placenta previa. Int J Gynaecol Obstet. 2006 Apr;93(1):51–2.

9. Carbillon L. [The therapeutic role of tocolysis]. J Gynecol Obstet Biol Reprod (Paris). 2004 Feb;33(1 Suppl):S45-50.

10. Towers C V, Pircon RA, Heppard M, C.V. T, R.A. P, M. H, et al. Is tocolysis safe in the management of third-trimester bleeding? Am J Obstet Gynecol [Internet]. 1999 Jun;180(6 I):1572–8. Available from: http://ovidsp.ovid.com/ovidweb.cgi?T=JS&PAGE=reference&D=emed7&NEWS=N&AN=29293286

11. Besinger RE, Moniak CW, Paskiewicz LS, Fisher SG, Tomich PG. The effect of tocolytic use in the management of symptomatic placenta previa. Am J Obstet Gynecol. 1995 Jun;172(6):1770–8.

12. W.J. W. Magnesium sulfate tocolysis in selected patients with symptomatic placenta previa. Am J Perinatol [Internet]. 1990;7(3):251–3. Available from: http://ovidsp.ovid.com/ovidweb.cgi?T=JS&PAGE=reference&D=emed5&NEWS=N&AN=20225264

13. D.N. SJ, D.A. N, M.J. P. Tocolysis in the management of third trimester bleeding. J Perinatol [Internet]. 1990;10(2):125–8. Available from: http://ovidsp.ovid.com/ovidweb.cgi?T=JS&PAGE=reference&D=emed5&NEWS=N&AN=20839454

14. Celayeta MA. Comment: Tocolysis in placenta previa. Vol. 22, Drug intelligence & clinical pharmacy. United States; 1988. p. 828.

15. Tomich PG. Prolonged use of tocolytic agents in the expectant management of placenta previa. J Reprod Med. 1985 Oct;30(10):745–8.

16. Nakamura Y, Nomura Y, Shinagawa S. Clinical usefulness of terbutaline in the management of placenta praevia. Nihon Sanka Fujinka Gakkai Zasshi. 1984 Jun;36(6):947–9.

17. M.B. S, O. L, A.M. T, J.L. T. Tocolysis with terbutaline sulfate in patients with placenta previa complicated by premature labor. J Reprod Med Obstet Gynecol [Internet]. 1984;29(4):248–50. Available from: http://ovidsp.ovid.com/ovidweb.cgi?T=JS&PAGE=reference&D=emed3&NEWS=N&AN=14138872

18. Flowers WK. Ritodrine use in placenta previa. J Natl Med Assoc. 1983 Apr;75(4):427–8.

19. Hastwell G, Lambert BE. A comparison of salbutamol and ritodrine when used to inhibit premature labour complicated by ante-partum haemorrhage. Curr Med Res Opin [Internet]. 1979;5(10):785–9. Available from: http://ovidsp.ovid.com/ovidweb.cgi?T=JS&PAGE=reference&D=med1&NEWS=N&AN=373993

20. Beta mimetic drugs in obstetrics. CLININVESTGINECOBSTET [Internet]. 1975;2(2):93–114. Available from: http://ovidsp.ovid.com/ovidweb.cgi?T=JS&PAGE=reference&D=emcl2&NEWS=N&AN=7038662

21. K.H. M, F. L, W. D. Tocolytic therapy in obstetrics. J Perinat Med [Internet]. 1974;2(1):3–16. Available from: http://ovidsp.ovid.com/ovidweb.cgi?T=JS&PAGE=reference&D=emcl2&NEWS=N&AN=5027562

22. G. C, V. S. Clinical use of a beta-mimetic drug in the control of uterine dynamics. Ann Ostet Ginecol Med Perinat [Internet]. 1973;94(9–10):587–94. Available from: http://ovidsp.ovid.com/ovidweb.cgi?T=JS&PAGE=reference&D=emed2&NEWS=N&AN=5531345

23. Bose DA, Assel BG, Hill JB, Chauhan SP. Maintenance tocolytics for preterm symptomatic placenta previa: a review. Am J Perinatol. 2011 Jan;28(1):45–50.

24. H. L, X. G. Expectant treatment of placenta previa with ritodrine. Zhonghua Fu Chan Ke Za Zhi [Internet]. 1998;33(4):204–5. Available from: http://ovidsp.ovid.com/ovidweb.cgi?T=JS&PAGE=reference&D=emed7&NEWS=N&AN=31288084

25. Besinger RE , Moniak CW , Paskiewicz LS FS and TP. The effect of tocolytic use in the management of symptomatic placenta previa. Am J Obstet Gynecol 1995, 172(6), 1770. 1995;172(6):1770.

**Appendix 3 Characteristics of included studies**

| **Verspyck et al 2017** (1) |  |
| --- | --- |
| methods | Individual randomisation using sealed opaque sequentially numbered envelopes |
| Participants | 109 pregnant women presenting to perinatal units including all tertiary centers in the North West of France, with a confirmed diagnosis of placenta praevia  Mean age: 31.6years in the nifedipine group, 30.6 years in the placebo group.  Inclusion criteria: women aged ≥ 18 years with a singleton fetus and intact membranes.  Exclusion criteria: severe hemorrhage  with immediate delivery required, pre-eclampsia, placental abruption, fetal distress, intrauterine  growth restriction, intrauterine fetal death, chorioamnionitis, liver disease, severe chronic  renal disease, heart disease, and contra indications for nifedipine. |
| Intervention | Allocation to receive tocolysis. Women were allocated to receive r 20 mg of slow-release nifedipine three times daily (60 mg) until 36 + 6 weeks of gestation.  Controls: The controls received placebo until 36 + 6 weeks of gestation |
| Outcomes | The primary outcome was prolongation of pregnancy  secondary outcomes (Maternal )   - bleeding recurrence, - need for blood transfusion, - immediate pre-delivery hemoglobin count, - cesarean delivery and indications, - post delivery hemoglobin count, - use of additional uterine devascularisation procedure or peripartum hysterectomy, - length of hospitalization and - death.   Neonatal secondary outcomes   - were term of birth, - birthweight, - Apgar score less than 7 at 5 minutes, - umbilical arterial pH <7.00, - hospitalization in neonatal intensive care unit, - length of hospitalization, - adverse neonatal outcomes associated with prematurity and - perinatal death. |
| Notes | All patients received routine care as was standard practice in the hospitals. |

**Risk of Bias table Verspyck et al 2017** **Outcome= days of pregnancy prolongation**

| **Domain** | **Review authors’ judgement** | **Support for judgement** |
| --- | --- | --- |
| **Random sequence generation**  **(selection bias)** | ***Low risk*** | The investigators describe a random component in the sequence generation and allocation using a computer random sequence generator. This should produce comparable groups.  Quotation... “Group assignment was stratified by center and based on a computer generated random sequence in balanced blocks (Department of Biostatistics Rouen, France)”.  Comment: sequence generation adequate |
| **Allocation concealment**  **(selection bias)** | ***Low risk*** | Participants and investigators enrolling participants could not foresee assignment because the allocation sequence was computer generated. Investigators and participants could not therefore have known the allocation sequence in advance or during enrolment.  Quotation... “Group assignment was stratified by center and based on a computer generated random sequence in balanced blocks (Department of Biostatistics Rouen, France)”.  Comment: allocation process adequate |
| **Blinding of participants and personnel**  **(Performance bias)** | ***Low risk*** | This is a placebo-controlled trial, implying the placebo helped to ensure blinding of participants and trial investigators. Throughout the trial, the investigators ensured that all those involved in the trial remained unaware of the intervention a participant received. It is therefore unlikely that this blinding was broken.  Quote from the article…“Treatment assignment and block size were blinded to investigators, participants, clinicians, and research nurses”.  Quote from the article...”Treatment allocation was never unblinded during the study”.  Comment: the authors did not specifically mention the placebo was identical to the intervention drug but this can be judged to be the case, given that participants and those involved in the trial remained blinded to the treatment the patients were receiving. |
| **Blinding of outcome assessment**  *(detection bias)* | ***Low risk*** | Outcome assessors and study participants remained blinded to the intervention arms of the participants  Quote from the protocol “The treatment period in parallel groups is double-blinded. The blind will be maintained until final data analysis.”  Quote from the article…“Treatment assignment and block size were blinded to investigators, participants, clinicians, and research nurses”.  Quote from the article...” Treatment allocation was never unblinded during the study”.  Comment: The primary outcome of days of pregnancy prolongation from randomisation is a hard outcome given that delivery is a hard outcome, which is not likely to be influenced by any lack of blinding (which was not the case). |
| **Incomplete outcome data**  **(Attrition bias)** | ***Low risk*** | There were no losses to follow up, no withdrawals, no missing data and no exclusions. All 109 patients who were randomised, were also included in the analysis of the primary outcome, and, analysis was on an intention to treat basis.  Figure 1 (page 6) of the article shows the flow chart of study participants. |
| **Selective reporting**  **(Reporting bias)** | ***Low risk*** | A protocol for the study is available and all expected outcomes are identified (Primary efficacy parameters prolongation of pregnancy; Secondary efficacy parameters, Delta hemoglobin level at inclusion and in the immediate postpartum period, the need for transfusion or prescription of Venofer, Birth weight, Term at birth, length of hospitalization, Morbidity, mortality), and reported as planned by the study.  Tables 1, 2 and 3 summarises all of this information. |
| **Other sources of bias** | ***Low risk*** | The study appears to be free of other sources of bias. |
|  |  | Overall, we conclude that this study was at low risk of bias for the primary outcome of pregnancy prolongation given that in the 7 domains, it was assessed as being of low risk of bias. |

| **Sharma et al 2004** (2) |  |
| --- | --- |
| Methods | Individual randomisation using Tippet’s random number table |
| Participants | 60 pregnant women presenting to the labor room with antepartum  hemorrhage with a diagnosis of placenta previa confirmed by ultrasound  Mean age: 27.17 years in the ritodrine group, 25.33 years in the placebo group.  Inclusion criteria were pregnant women with any type of symptomatic  placenta previa diagnosed by transabdominal ultrasonography and were included if their gestational age was within 28–34 weeks at the time of  admission, HbG9 g% and had a mild degree of bleeding  Exclusion criteria included moderate or severe bleeding, labor, known case of diabetes gestational diabetes mellitus, severe pre-eclampsia,  chronic hypertension, heart disease, liver disease, chronic renal disease, or fetal distress/intrauterine fetal death |
| Interventions | Allocation to receive tocolysis. “The tocolysis was started with administration of ritodrine in the study group as intramuscular injections in dosage of 10 mg every 6 h for 7 days.”  Controls: “The control group did not receive tocolysis” |
| Outcomes | The primary outcome was prolongation of pregnancy,  Secondary outcomes   - number of episodes of bleeding, - amount of bleeding, - number of blood transfusions required and - neonatal outcome. |
| Notes | Additional trial data not reported was provided by the authors |

**Risk of Bias assessment Sharma et al 2004: Outcome= days of pregnancy prolongation**

| Domain | Risk of bias | | | Support for judgement  *(include direct quotes where available with explanatory comments)* | Location in text or source *(pg & ¶/fig/table/other)* |
| --- | --- | --- | --- | --- | --- |
|  | Low | High | Unclear |  |  |
| Random sequence generation *(selection bias)* | ☑ |  |  | The investigators describe a random component in the sequence generation and allocation using the Tippet’s random number table. This should produce comparable groups.  Quotation from article.. “A total number of 60 women were recruited and randomly allocated to the two study groups using Tippet’s random number table”. | P110 |
| Allocation concealment *(selection bias)* | ☑ |  |  | Allocation was concealed in advance given that participants were randomly allocated using the Tippet’s random number table.  Quotation from article.. “A total number of 60 women were recruited and randomly allocated to the two study groups using Tippet’s random number table”. | P110 |
| Blinding of participants and personnel *(performance bias)* |  | ☑ |  | Participants and personnel were not blinded as the intervention was given intramuscularly, and the control group did not receive any placebo. This lack of blinding could potentially affect attention given to patients.  Quotation.. “The tocolysis was started with administration of ritodrine in the study group as intramuscular injections “…  “The control group did not receive tocolysis” | P110 |
| Blinding of outcome assessment *(detection bias)* | ☑ |  |  | Outcome assessors and study participants were not blinded to the intervention arms of the participants  Quotation.. “The tocolysis was started with administration of ritodrine in the study group as intramuscular injections “…  “The control group did not receive tocolysis”  Comment: The primary outcome of days of pregnancy prolongation from randomisation is a hard outcome given that delivery is a hard outcome, which is not likely to be influenced by any lack of blinding. | P.112 |
| Incomplete outcome data *(attrition bias)* | ☑ |  |  | There were no losses to follow up, no withdrawals, no missing data and no exclusions. All 60 patients who were randomised were also included in the analysis of the primary outcome. See Table 3, P. 112. | P 112 |
| Selective outcome reporting? *(reporting bias)* | ☑ |  |  | A protocol for the study is not available but all expected outcomes identified in the methods (Primary efficacy parameters prolongation of pregnancy; Secondary efficacy parameters, change in hemoglobin, random blood sugar and serum electrolyte), are reported as planned by the study.  Tables 2 and 3 summarises all of this information | P110, P112 |
| Other bias | ☑ |  |  | The study appears to be free of other sources of bias. |  |
| Notes: Overall, we conclude that this study was at low risk of bias for the primary outcome of pregnancy prolongation given that in 6 of the 7 domains, it was assessed as being of low risk of bias. The non-blinding of study personnel and participants even though a potential source of bias, is unlikely to outweigh the overall low risk of bias of the study. | | | | | |

| **Sozanski 1985** (3) |  |
| --- | --- |
| Methods | No mention or randomisation method |
| Participants | 84 Pregnant women with ultrasonically recognized placenta previa  Age range in treatment group: 19-39 years; age range in control group: 21-43 years  Inclusion criteria: women aged ≥ 19 years with ultrasonically recognized placenta praevia between 21 to 36 weeks pregnancy  Exclusion criteria: Not stated |
| Intervention | Allocation to receive tocolysis. Intervention group received pertusistens (fenoterol) and additional lytic cocktail  Control group received same additional drug cocktail as the treatment group; but did not receive treatment drug: partusisten. |
| Outcome | The primary outcome was prolongation of pregnancy. |
| Notes | Only provide mean gestational age at delivery and length of hospital stay information for 6 cases of greatest prolongation of duration in the treatment group – this data was not available for all 84 women and there is no comparison made either |
|  |  |

**Risk of Bias assessment Sozanski 1985** **Outcome= days of pregnancy prolongation**

| Domain | Risk of bias | | | Support for judgement  *(include direct quotes where available with explanatory comments)* | Location in text or source *(pg & ¶/fig/table/other)* |
| --- | --- | --- | --- | --- | --- |
|  | Low | High | Unclear |  |  |
| Random sequence generation *(selection bias)* |  |  |  | High – no mention of randomization at all |  |
| Allocation concealment *(selection bias)* |  |  |  | High – no mention of randomization at all |  |
| Blinding of participants and personnel *(performance bias)* |  |  |  | Outcome group: All/  Control group received relanium and same additional drug cocktail as the treatment group; but did not receive treatment drug: partusisten. No explicit mention about blinding. |  |
| *(if separate judgement by outcome(s) required)* |  |  |  | Outcome group:  NA |  |
| Blinding of outcome assessment *(detection bias)* |  |  |  | Outcome group: All/  Based on your comments in our email communication - since the outcome is pregnancy prolongation defined by time of delivery, blinding may not be important for outcome assessment, therefore low risk of bias. |  |
| *(if separate judgement by outcome(s) required)* |  |  |  | Outcome group:  NA |  |
| Incomplete outcome data *(attrition bias)* |  |  |  | Outcome group: All/  Cannot compare number randomized to number analysed, but it appears that all the women they enrolled were followed-up. No mention of any loss to follow-up or adverse events. |  |
| *(if separate judgement by outcome(s) required)* |  |  |  | Outcome group:  NA |  |
| Selective outcome reporting? *(reporting bias)* |  |  |  | No trial registry to compare if pre-specified outcomes were all reported, but this trial only reported on 1 outcome: pregnancy prolongation. |  |
| Other bias |  |  |  | Funding = not reported |  |
| Notes: | | | | | |

References

1. E. V, I. C-L, M. D, P. D. Maintenance nifedipine therapy for preterm symptomatic placenta previa: The PPADAL randomized, multicenter, double-blind, placebo-controlled trial. Am J Obstet Gynecol [Internet]. 2015;212(1 SUPPL. 1):S15. Available from: http://ovidsp.ovid.com/ovidweb.cgi?T=JS&PAGE=reference&D=emed17&NEWS=N&AN=71742474

2. Sharma A, Suri V, Gupta I. Tocolytic therapy in conservative management of symptomatic placenta previa. Int J Gynaecol Obstet. 2004 Feb;84(2):109–13.

3. Sozanski L, Gerber J. [Effect of partusisten administered to pregnant women with uterine hemorrhage caused by placenta praevia on the prolongation of pregnancy]. Ginekol Pol. 1985 Dec;56(12):754–8.

| Outcome № of participants (studies) | Relative effect (95% CI) | **Anticipated absolute effects (95% CI)** | | | Certainty |
| --- | --- | --- | --- | --- | --- |
|  |  | **Without Tocolysis** | **With Tocolysis** | **Difference** |  |
| Pregnancy prolongation № of participants: 253 (3 RCTs) | - | The mean pregnancy prolongation was 22.2 Days | - The mean pregnancy prolongation was 33.9 Days | MD **11.51 Days more** (1.75 fewer to 24.76 more) | ⨁⨁◯◯ LOW ^a,b^ |
| Gestational age at delivery № of participants: 169 (2 RCTs) | - | The mean Gestational age at delivery was 34.9 weeks | - The mean Gestational age at delivery was 35.3 weeks | MD **0.33 Weeks higher** (1.53 lower to 2.19 higher) | ⨁⨁⨁◯ MODERATE ^c^ |
| Birth Weight № of participants: 169 (2 RCTs) | - | The mean birth Weight was **2.29** Kg | - The mean birth Weight was 2.42 Kg | MD **0.12 Kg higher** (0.26 lower to 0.5 higher) | ⨁⨁⨁◯ MODERATE ^c^ |
| Proportion of women with premature deliveries № of participants: 169 (2 RCTs) | **RR 1.04** (0.56 to 1.94) | 62.4% | **64.8%** (34.9 to 100.0) | **2.5% more** (27.4 fewer to 58.6 more) | ⨁⨁◯◯ LOW ^d^ |
| Repeat episodes of vaginal beeding № of participants: 169 (2 RCTs) | **RR 1.05** (0.73 to 1.51) | 62.4% | **65.5%** (45.5 to 94.2) | **3.1% more** (16.8 fewer to 31.8 more) | ⨁⨁◯◯ LOW ^d^ |
| Risk difference in Perinatal deaths № of participants: 169 (2 RCTs) | **0.0 --** (-0.04 to 0.03) | 1 perinatal death in 85 deliveries | **0 perinatal deaths in 84 deliveried** | **1.2% fewer** (1.2 fewer to 1.1 fewer) | ⨁⨁◯◯ LOW ^e^ |
| Duration of maternal hospitalisation № of participants: 109 (1 RCT) | - | 6.5 (SD 3.5) days | - 7.1 (SD 3.9) days | MD **0.6 higher** (0.79 lower to 1.99 higher) | ⨁⨁◯◯ LOW ^f^ |

**Appendix 4: Summary of Findings table of main outcomes tocolysis vs no tocolysis in symptomatic placenta praevia**

a. One trial (Gerber et al) was at high risk of bias given the absence of randomisation. A sensitivity analysis with and without this will alter our conclusions based on a minimally important difference of 7 days pregnancy prolongation for tocolysis ( MD 11.51 [-1.75, 24.76] and MD 5.46 [-4.86, 15.78] ). We therefore downgraded by 1 level.

b. The confidence interval of the final estimated mean difference of 11.51 [-1.75, 24.76] was very wide and crossed the threshold of benefit but did not cross the threshold of harm of 07 days. We therefore downgraded by 1 for this.

c. Considering the rule of thumb optimal information size of 400 participants for continuous outcomes, our total sample size of 169 patients is very small. We therefore downgraded by 1 levels for this.

d. The 95% confidence interval includes a substantial benefit to substantial harm, hence the estimate is imprecise. We therefore downgraded by 2 levels for this.

e. Considering the rule of thumb optimal information size of 300 events for dichotomous outcomes, our total number of just one perinatal death is very small. We therefore downgraded by 2 levels for this.

f. Considering the rule of thumb optimal information size of 400 participants for continuous outcomes, our total sample size of 129 patients is very small. This is an estimate from a single study. We therefore downgraded by 2 for this.

**Appendix 5 Sensitivity analysis**

***
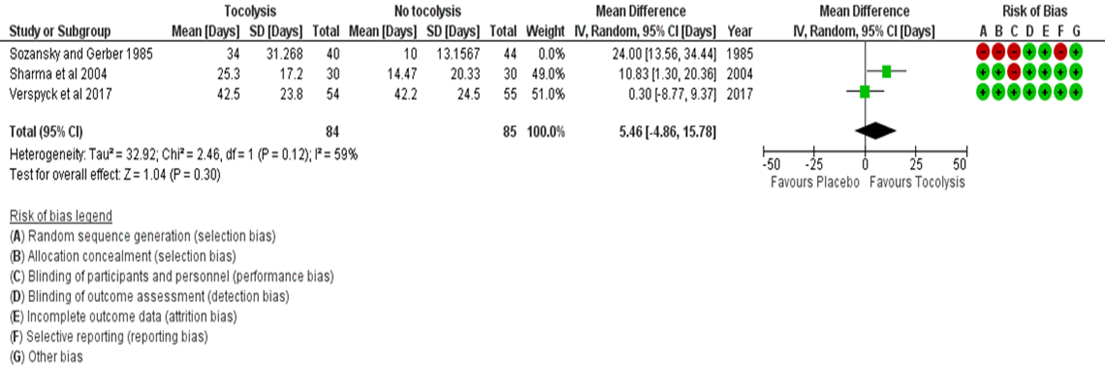
***

***Sensitivity analysis without study by Sozansky and Gerber considered at high risk of bias. Outcome: mean difference in number of days of pregnancy prolongation***
